# Rapamycin and mTOR: a serendipitous discovery and implications for breast cancer

**DOI:** 10.1186/2001-1326-1-29

**Published:** 2012-11-15

**Authors:** Belinda Seto

**Affiliations:** 1 National Institutes of Health, National Institute of Biomedical Imaging and Bioengineering, 9000 Rockville Pike, Building 31, Room 1C14, Bethesda, MD 20892, USA

**Keywords:** Rapamycin, Mammalian Target of Rapamycin (mTOR), Breast cancer, Targeted chemotherapeutics, Clinical translation

## Abstract

Rapamycin was discovered more than thirty years ago from a soil sample from the island of Rapa Nui. It was isolated from Streptomyces hygroscopicus and initial characterization focused on its antifungal activities. Subsequent characterization showed that it has immunosuppressive properties and has been used successfully to reduce organ rejection with kidney transplantation. Rapamycin has proven to be a versatile compound with several seemingly unrelated properties, including antifungal, immunosuppressive, and anticancer. The National Cancer Institute (NCI) Developmental Therapeutics Program demonstrated that rapamycin inhibited cell growth in tumor cell lines. These observations stimulated research to explore the underlying mechanism of anti-tumor activities. Cell growth inhibition involves binding to the mammalian Target of Rapamycin (mTOR). The mTOR signaling pathway is critical to cell growth, proliferation, and survival and rapamycin inhibits these hallmark processes of cancer. Binding of growth factors activates mTOR signaling, which in turn leads to downstream phosphorylation of protein kinases, e.g., p70S6 kinase and lipid kinases in the phosphorylation of phosphoinositides. Understanding of mTOR signaling provided the biological basis for targeted chemotherapeutics development, including several rapamycin analogues for treating breast and other cancers.

## Review

### Introduction

Breast cancer is the second most commonly diagnosed cancer, after skin cancer, among U.S. women. In 2012, 227,000 new cases have been reported [1]. Recent developments in computed tomography imaging have improved the early detection of breast cancer, when treatment is most effective [2]. Concomitant with the technological development is the explosion of research findings on the molecular mechanisms of breast cancer. As a result, mechanism-based approaches have become increasingly used as strategies for therapeutic developments. This confluence of technology development in early diagnosis and improved therapeutics has led to a decline in breast cancer death in recent years, although death rates are still higher than all types of cancer other than lung cancer [3].

This report describes a tale of discovery that reinforces the serendipitous nature of basic research and the notion that discoveries may lead to unanticipated outcomes in other disciplines. In this particular story, the isolation of the bacterium Streptomyces hygroscopicus from a soil sample three decades ago on a remote island led to intense, multifaceted research that changed the way breast cancer is treated. The identification of rapamycin from Streptomyces hygroscopicus as an antifungal agent, through being an immune inhibitor to being an effective anticancer drug, demonstrates a research continuum driven by clinical observations that were critical in the elucidation of the mTOR pathway. Rapamycin provided the stimulus for research on the complex and pivotal mTOR pathway that transmits signals through which it controls a range of vital biological processes. The dissection of the molecular networks of interacting signaling pathways has led to improved understanding of the transcription, protein synthesis, and metabolic processes that underpin oncogenic transformation. Such knowledge has led to therapeutic developments that yielded targeted drugs for breast cancer patients. For patients who are estrogen and/or progesterone receptor positive, endocrine therapies offer treatments that interfere with the signaling pathway involved in cell growth and proliferation. Two targeted therapeutic examples include aromatase inhibitors, which interfere with estrogen production, and tamoxifen, which interferes with estrogen binding to the receptor. For patients who are HER-2 positive, targeted therapies with HER2 antibodies, such as trastuzumab and lapatinib, offer possible treatment options [4].

This review will focus on the mammalian Target of Rapamycin (mTOR) pathway and also provide a perspective on translational research, from the chemical and pharmacologic characterization of rapamycin to the molecular mechanisms of breast cancer, ending with clinical applications and treatments.

### Discovery of rapamycin

Rapamycin, (also known by its generic name, Sirolimus) is a natural product isolated from Streptomyces hygroscopicus, found on the island of Rapa Nui in 1972 [5]. Structural studies showed that it is similar to an antibiotic FK506 [6], a macrolide lactone. Studies following its discovery showed rapamycin to exhibit multiple properties, including antibacterial activity, antifungal (anti-Candida), and immunosuppressive effects. It inhibits antigen-induced T cell and B cell proliferation and antibody formation. The latter finding has significant clinical implications as rapamycin was developed into an immunosuppressant drug for patients following organ transplantation. It was approved by the U.S. Food and Drug Administration (FDA) as a prophylaxis for renal rejection. Wyeth Pharmaceuticals marketed Rapamune as an immunosuppressant for use in conjunction with corticosteroids and cyclosporine to prevent kidney rejection [7].

The discovery that rapamycin was an immunosuppressant might not have led to testing its potential as a viable tumor suppressor if it were not for the research of Dr. Suren Sehgal at Ayerst Research Laboratories, Montreal, where rapamycin was isolated in 1972. Intuitively one would have thought that an immunosuppressive compound would prevent an immune response against tumor cells and therefore would not be a likely anticancer drug. But Dr. Sehgal observed that this compound appeared to possess novel properties beyond its immunosuppressive activities [8]. He sent a sample of rapamycin to the National Cancer Institute (NCI) Developmental Therapeutics Program and requested anti-tumor activity screening. As a standard screening protocol, NCI initially tested compounds for growth inhibition against a limited number of human tumor cell lines. If the compound showed inhibition against one of more of these cell lines, it would be further tested for growth inhibition or killing of one or more of the NCI standard 60 human tumor cell lines with varying concentrations of the compounds. Approximately 2% of the 2500 compounds tested annually proceed to the next stages of in vivo tests in xenographs in mice. Against the 60 tumor cell line panel, rapamycin was found to inhibit the growth of a number of tumor cell lines including mammary, colon 26, B16 43 melanocarcinma, and EM ependymoblastoma [9]. Based on these test results, NCI advanced rapamycin as a priority drug.

### Mammalian Target of Rapamycin (mTOR)

Following the NCI finding of anti-tumor activities in rapamycin, numerous reports were published confirming its inhibitory effect on cell growth [10,11] in several organisms: Saccharomyces cerevisiae [12], Drosophila [13,14], Caenorhabditis elegans [15], fungus [16], plants [17], and mammals [18]. In these organisms, the inhibitory mechanism involves binding to the target proteins, collectively named Target of Rapamycin (TOR) [12]. The specifics of the inhibitory mechanisms differ with the various organisms. However, there are consistent observations that these proteins are highly conserved evolutionarily [19]. TOR protein sequences from eukaryotes share approximately 40% to 60% homology and several structural motifs are conserved [20]. Human TOR protein showed even higher homology in the primary sequence with other mammalian TORs (mTOR), 95% [21].

Biochemical studies showed that mTOR forms two complexes: mTORC1 and mTORC2 [22]. The mTORC1 complex is composed of mTOR, Raptor (regulatory-associated protein of mTOR) and mLST8/GβL (mammalian lethal with Sec13protein8/G-protein β-subunit-like protein), PRAS_40_ (proline-rich AKT substrate of 40kDa) and DEPTOR (DEP-domain-containing mTOR-interacting protein) [23]. mTORC1 is the catalytic kinase complex. The component proteins of mTORC1 contain a large number of conserved motifs, including those that are essential for protein-protein interactions. This observation, together with the finding of kinase activity in this complex, suggests that this complex may be the nexus for mTOR signaling. Rapamycin does not inhibit the mTORC1 kinase activity directly. It initially forms a complex with the FK506 binding protein (FKPB_12_), which in turn binds mTORC1 [24-27].

The observation that TOR proteins are conserved across the broad spectrum of organisms, from simple eukaryotes to mammals, led to subsequent investigations of TOR functions. The assumption that TORs might play a vital role in survival is well-grounded, as these proteins, with some variations structurally, are conserved through evolution. Thus, it is concluded that TOR functions are not only fundamental to survival, but that they afford evolutionary advantages. For its vital role in cell survival, the TOR pathway receives signaling inputs from insulin, growth factors and nutrients. The TOR pathway is central to regulating cell growth (cell size or mass) and proliferation (cell number), and responding to stress such as nutrient starvation (glucose or amino acids) that ultimately affects cellular energy levels [13,14,28,29]. Underpinning mTOR’s involvement in cell growth are the associated processes including transcription, protein translation, and cell cycle regulation from G1 to S phase. Given the importance of these biological processes, it should come as no surprise that the TOR pathway is involved in many disease processes. While a comprehensive review of mTOR’s role in disease processes is beyond the scope of this report, it is important to understand the mTOR signaling mechanism as it underlies many disease processes and has served to guide cancer therapeutic development and treatment.

### mTOR signaling pathway and regulatory network

**Figure 1:**
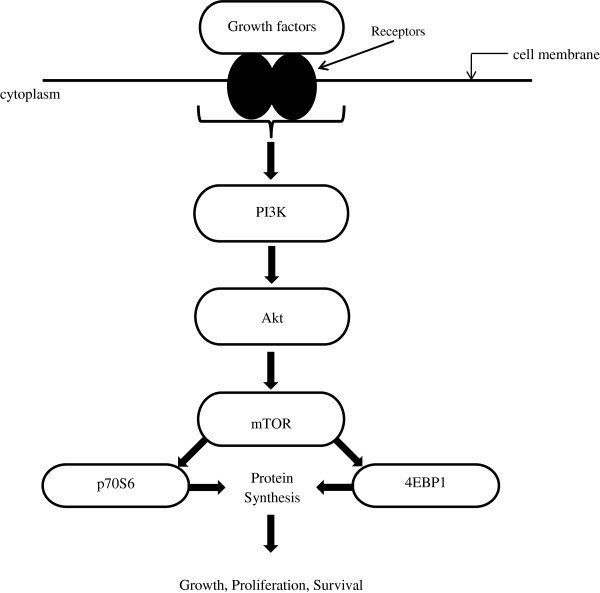
mTOR pathway.

**Figure 2:**
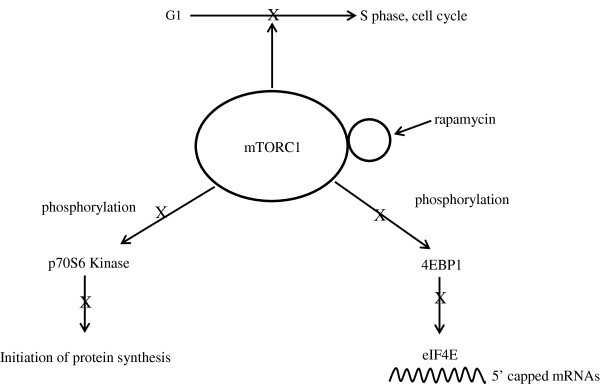
Rapamycin inhibition.

mTOR is a serine/threonine kinase of the phosphatidylinositol 3-kinase-related kinase family, and it is regulated through the PI_3_K and Akt/PKB pathway [30]. Growth factors (insulin-like growth factor 1 (IGF-1), insulin, epidermal growth factor, vascular epithelial growth factor) when bound to the cell surface receptors, activate the intracellular signaling of the PI_3_K/Akt pathway (Figure 1). The downstream effect of this activation is the phosphorylation of p70S6 Kinase and 4EBP1 [31]. An additional downstream effect is an increased phosphorylation of serine_2448_ on mTOR. Indication that mTOR signaling is involved in oncogenic transformation stemmed from studies of Akt mutants with kinase activity but failed to phosphorylate p70S6 kinase and 4EBP1. These mutants failed to transform chicken embryo fibroblast cells [32]. mTOR-driven phosphorylation of key proteins is an intricate balance of regulatory switches that determine which mRNA will be translated as a result of mTOR kinase activity. For example, mTOR phosphorylation of p70S6 kinase leads to downstream phosphorylation of the 40S ribosomal protein S6, resulting in increased translation from mRNAs that contain the 5^′^-terminal oligopyrimidine tract, such as those for the elongation factor-1α [33]. Together, these steps lead to increased ribosomal biosynthesis and protein synthesis generally. Activation of 4EBP1 translation initiation factor, on the other hand, leads to increased translation from mRNAs with 5^′^-untranslated regions such as those for cyclin D1 and *cmyc*, which are crucial to cell cycling (Figure 2). These examples illustrate mTOR’s role in regulating protein biosynthesis by phosphorylating key proteins.

Another important process that is regulated by PI_3_K signaling involves lipid kinases in the phosphorylation of phosphoinositides. Activated PI_3_K leads to increased production of phosphatidylinositol 3, 4, 5-triphosphate (PIP_3_), which in turn recruits Akt for cell growth, proliferation, and survival. These are hallmarks for cancers. Conversely, PIP_3_ is negatively regulated by a tumor suppressor, phosphatase and tensin homolog (PTEN) via dephosphorylation. Phosphorylation is also inhibited by rapamycin. It should come as no surprise, due to the significance of the regulatory activities of the PI_3_K/Akt pathway and its interaction with mTOR, that dysfunction of these signaling activities would alter cellular functions, as observed in most cancers. Dysfunction can also stem from genetic mutations. Mutations or gene amplification are found in components of the PI_3_K/Akt pathway in a large number of tumors [34]. A remarkably large percentage of breast cancer, greater than 70%, was found to have mutations in the genes involved in this pathway [35].

### mTOR inhibitors target breast cancer mechanism

Recognition of rapamycin’s anti-tumor target of the mTOR pathway led to the development of analogues of rapamycin as chemotherapeutic agents against solid tumor types, including breast cancer. However, there are substantial challenges with the pharmacokinetics of rapamycin due to its lipophilic chemistry [32]. Various formulations have been tested to improve its poor water solubility and bioavailability for clinical applications. Currently three analogues of rapamycin have been developed: Temsirolimus (Wyeth/Pfizer) [36], Deforolimus or Ridaforolimus (Merck/Ariad) and Everolimus, manufactured by Novartis [35,37,38]. Although these analogues differ in their formulation and bioavailability, the mechanism of inhibition is the same, binding to the mTORC1 target, thereby arresting cell cycling at the G1 phase. Temsirolimus was approved by the FDA for treating renal cell carcinoma. For metastatic breast cancer, temsirolimus in combination with letrozole was used in a phase III trial, but the combination of drugs did not show benefit over letrozole (aromatase inhibitor) alone [39].

As an mTORC1 inhibitor, everolimus reduces Akt and mTOR signaling, resulting in increased apoptosis. Everolimus alone or in combination with tamoxifen has been evaluated in postmenopausal breast cancer patients with hormone receptor positive, HER2 negative metastatic breast cancer. Bachelot et al. [40] reported a 46% reduction in risk of progression with the combination of tamoxifen and everolimus vs. tamoxifen alone. Risk of death was also reduced, by 55%, in the combined drug treatment group. For patients whose disease has progressed despite treatment with trastuzumab and chemotherapy, it has been shown that the addition of everolimus to trastuzumab and chemotherapy provides a 19% to 44% response rate [41-43]. A summary of the therapeutic compounds discussed in this review is listed in Table 1.

**Table 1:** Summary of cancer drugs in this review

**Rapamycin analogues**	**Known target**	**Disease**
Temsirolimus	mTOR	Renal cell carcinoma
Ridaforolimus (formerly known as Deforolimus)	mTOR	Breast cancer
		Soft tissue sarcoma
		Head and neck cancer
		Non-small cell lung cancer
		Colorectal cancer
Everolimus	mTOR	Hormone receptor positive, HER2 negative breast cancer
Inhibitors/Antibodies		
Letrozole	Competitive inhibitor of aromatase	Breast cancer
Tamoxifen	Antagonist of estrogen receptor	Breast cancer
Trastuzumab	HER2 receptor	Breast cancer
Aromasin	Aromatase inhibitor	Hormone receptor positive, HER2 negative breast cancer
Lapatinib	EGFR	HER2 positive breast cancer

It is postulated that there are cross-talks between signaling pathways: hormone signaling and the PI3K/Akt/mTOR pathways. Hormone receptor positive tumors rely on hormone-mediated signaling for growth. However, as hormone treatment continues, adaptive upregulation of growth factor mediated signaling, such as the PI3K/Akt/mTOR pathway, reinforces cross-talks leading to constitutive activation of the cell growth pathways, rendering the patients resistant to hormone treatment [35,44-47]. Everolimus, by inhibiting the PI3K/Akt/mTOR signaling, has been shown to restore hormone sensitivity [48]. Everolimus was recently approved by the FDA for use in combination with Aromasin for treating advanced hormone-receptor positive HER2-negative breast cancer [49].

### Translational research: a public-private partnership

The discovery of rapamycin in 1972 was serendipitous but this fortuitous beginning has led to immense impact on medicine. Over the subsequent decades, its activities have been widely investigated. It has been found to be an exceptionally “versatile” molecule in that it possesses antifungal, immunosuppressive, and anticancer activities. These characteristics attracted investigators from different disciplines to pursue basic research on the pharmacology of rapamycin, synthetic chemistry to produce analogues, mechanistic studies on disease processes, and clinical research on therapeutic development and disease treatment. However, a single individual, Dr. Suren Sehgal, is noteworthy for his keen observation that rapamycin may have antitumor activities. He contacted the NCI to test rapamycin in order to confirm his suspicion. His research was made even more poignant as his employer made a management decision that practically shut down his research on rapamycin. After several years of inactivity, rapamycin research was resurrected when Wyeth and Ayerst merged and the company leadership was convinced by the promising results from animal testing to continue funding rapamycin therapeutic development.

The NCI’s Developmental Therapeutics Program [50] was established by Congress in 1955 as the Cancer Chemotherapy National Service Center to provide preclinical data on compounds that the NCI solicits from government laboratories, academic institutions, and industry [49]. These compounds include both synthetic chemicals (140,000) and natural products (80,000). Of all the compounds screened by this program, approximately 40% originated from industry. Research from DTP has led to anticancer drugs that are in use today. For example, Paclitaxel was discovered as a natural product from Yew trees and developed for clinical use for breast and other types of cancer [9]. Recently, DTP research led to the development of eribulin mesilate as a microtubule inhibitor for metastatic breast cancer [51], and FDA approval was issued in 2010. DTP has been successful in producing more than 40 U.S. licensed chemotherapeutic drugs. Many of these have been produced in collaboration with the commercial sector. The Everolimus clinical trial sponsored by Novartis is the translational product of privately- and federally-funded basic research on rapamycin and the PI3K/Akt/mTOR signaling. This is another example that illustrates the partnership between the federal government and the industrial partners that is the cornerstone of clinical translational research.

Another NIH program that facilitates small molecule screening is the Molecular Library Screening Centers Network (MLSCN), established in 2008, to provide large-scale screening capacity necessary to identify small molecules that can be optimized as chemical probes to study the functions of genes, cells, and biochemical pathways in health and disease. These small molecules may be used by researchers in the public and private sectors to validate new drug targets, which could then move into the drug-development pipeline. The first example of successful translation resulting from small molecule screening to Phase I clinical trial was the identification of Sphingosine-1-phosphate receptor. This receptor and related molecules were identified from research conducted by the Scripps Research Institute as part of the molecular library program. These small molecules were further developed by Scripps and a private entity, Receptors, Inc., for potential treatment for multiple sclerosis [52]. It is hoped, through continuing collaborations among preclinical and clinical investigators in both the public and private sectors, that breast cancer therapeutics will continue to be developed based on the molecular mechanism of the disease. The enhancement of the armamentarium for breast cancer should continue to reduce the mortality and morbidity for patients.

## Conclusion

The story of rapamycin illustrates the need for basic discovery research and the elucidation of biological mechanisms to inform translation to clinical research and clinical trials. It may take decades to unravel the full complexity of biological systems. Basic and translational research is typically funded by the government. However, there is an important role for public-private partnership in research, especially as it advances to clinical trials as described in this report.

## Competing interests

The author declares that she has no competing interests.
